# MALDI-TOF mass spectrometry profiling of bovine skim milk for subclinical mastitis detection

**DOI:** 10.3389/fvets.2022.1009928

**Published:** 2022-12-01

**Authors:** Matteo Cuccato, Sara Divari, Paola Sacchi, Flavia Girolami, Francesca Tiziana Cannizzo

**Affiliations:** Department of Veterinary Sciences, University of Turin, Turin, Italy

**Keywords:** bovine milk, MALDI-TOF mass spectrometry, mastitis, somatic cell counts, protein profile

## Abstract

**Introduction:**

Mastitis is one of most impacting health issues in bovine dairy farming that reduces milk yield and quality, leading to important economic losses. Subclinical forms of the disease are routinely monitored through the measurement of somatic cell count (SCC) and microbiological tests. However, their identification can be tricky, reducing the possibilities of early treatments. In this study, a MALDI-TOF mass spectrometry approach was applied to milk samples collected from cows classified according to the SCC, to identify differences in polypeptide/protein profiles.

**Materials and methods:**

Twenty-nine raw milk samples with SCC >200,000 cell/ml (group H) and 91 samples with SCC lower than 200,000 (group L) were randomly collected from 12 dairy farms. Spectral profiles from skim milk were acquired in the positive linear mode within the 4,000–20,000 m/z mass acquisition range.

**Results and discussion:**

Based on signal intensity, a total of 24 peaks emerged as significant different between the two groups. The most discriminant signals (4,218.2 and 4,342.98 m/z) presented a ROC curve with AUC values higher than 0.8. Classification algorithms (i.e., quick classifier, genetic algorithm, and supervised neural network) were applied for generating models able to classify new spectra (i.e., samples) into the two classes. Our results support the MALDI-TOF mass spectrometry profiling as a tool to detect mastitic milk samples and to potentially discover biomarkers of the disease. Thanks to its rapidity and low-cost, such method could be associated with the SCC measurement for the early diagnosis of subclinical mastitis.

## Introduction

Mastitis is the most frequent and detrimental disease in dairy cows. The inflammation of the udder during lactation is the major cause of economic losses in the dairy industry, due to the reduction in milk production and quality, the increased costs for treatments, and the grown herd turnover (1, 2). It is a complex multi-etiological disease caused by a variety of microorganisms, mainly bacteria (*Staphylococcus* spp., *Streptococcus* spp., and *Enterobacteriaceae*). Bovine mastitis is classified into clinical and subclinical forms according to the presence or absence of symptoms and signs, such as visibly abnormal milk, and swelling, heat, pain and redness of the udder (3). Subclinical mastitis is very challenging and difficult to diagnose, because of the normal appearance of both mammary gland and milk; the only indicators of infection are the increased somatic cell count (SCC) and the bacterial population in milk (4). According to a recent systematic review and meta-analysis of the prevalence of bovine mastitis worldwide, the subclinical forms of the disease are the most prevalent and cause the major loss of milk production (5, 6). Therefore, the early detection of subclinical mastitis is crucial for an effective treatment and the successful dairy herd management.

More to the point, in the European Union the recent implementation of Reg. EU No. 6/2019 about veterinary medicinal products has strictly limited the use of antimicrobials for prophylaxis and metaphylaxis purposes to prevent the insurgence and spread of resistance phenomena. In the last decades, the use of antibiotics for the preventive control of possible outbreaks of mastitis has been regularly adopted, especially during the dry period (2, 5). In the new regulatory scenario, the veterinary practitioners acting in the dairy industry need alternative solutions for the control of mastitis, including novel diagnostic and therapeutic tools.

The routinely used methods to diagnose the subclinical forms of mastitis are the measurement of SCC and the microbiological test (3). The SCC level is also one of the parameters to assess milk suitability for human consumption, therefore influencing milk pricing (7). It is widely accepted that mastitic milk has a SCC value higher than 400,000 cells/ml, regardless of the presence of clinical symptoms (1, 8). On the contrary, the milk collected from a healthy mammary gland is characterized by a SCC value lower than 100,000 cells/ml (1, 7). A SCC measurement comprised between those boundaries should be interpreted: it could be suggestive of a subclinical mastitis or the result of the recovering phase of udder infection, when inflammation and microbial pathogens could still be detected (8, 9). Currently, a SCC measurement equal to 200,000 cells/mL has been set as a threshold to classify subclinical mastitis (10). However, inflammation of the mammary gland has been also observed at values around 100,000 cells/mL, especially in primiparous cows (11).

In addition to SCC, another parameter that could be investigated and correlated to mammary gland inflammation is the protein composition of milk (12). During mastitis, milk proteins are enriched by host immune proteins, such as cytokines, acute phase proteins, chemokines, and lactoferrin, but also by bacterial enzymes, mainly proteases (12, 13). Several studies have explored the potentiality of protein biomarkers as alternative/integrative diagnostic tools by means of different approaches (13–16). Matrix-assisted laser desorption/ionization–time of flight mass spectrometry (MALDI-TOF MS) is frequently employed in human clinical studies for discovering protein markers associated with specific pathologies (e.g., cancer and neurodegenerative diseases) (17–19). The application to easily accessible biological specimens, such as plasma, saliva and urine, is especially promising for the untargeted biomarker search based on profiling pattern (18). Such an approach has been applied in a number of cases also to milk, mainly to detect food adulterations (20, 21).

In this study, we collected milk samples from cows reared in different dairy farms of the Piedmont Region (Italy) and classified them according to standard SCC measurement and microbiological analysis. The skim milk was then subjected to MALDI-TOF MS peptide/protein profiling. The main objective was to detect significant differences in the milk proteome pattern correlated to the SCC values, providing a new tool for both the early diagnosis of bovine mastitis and the discovery of biomarkers of the disease.

## Materials and methods

### Sample collection and mastitis screening tests

One-hundred and twenty primiparous Holstein Friesian cows belonging to 12 dairy farms located in the provinces of Cuneo and Turin (Piedmont Region, Northern Italy) were selected. Animals were managed according to the local farm-production practices. All manipulations were performed kindly to avoid animal distress. Before milk collection, teats were disinfected, and the first squirts were collected in a specific container and discarded. Individual samples were collected in sterile polypropylene tubes from all quarters of each cow and immediately refrigerated. One aliquot was transferred to the A.R.A.P. laboratory (Associazione Regionale Allevatori, Cuneo, Italy) for the routine follow up of milk quality through functional feature analyses (i.e., SCC measurement and bacteriological examination). The SCC measurement was performed with the Fossomatic 7DC (Foss Italia, Padua, Italy) according to the certified protocol (ISO 13366-2 / IDF 148-2:2006). Bacterial culture through non-selective conditions and subsequent confirmation tests were performed according to the A.R.A.P. laboratory routine methods.

Milk aliquots for the proteomic investigation were defatted by centrifugation at 3,000 g for 10 min at + 4°C and the skimmed milk was stored at −80°C until further analysis. Supplementary Table 1 summarizes the information about the period of lactation of each cow and the results of the mastitis screening tests. For the proteomic analysis, the samples were divided into two groups based on the SCC value: group L includes milk samples with SCC lower than 200,000 cells/ml (*n* = 91) and group H includes milk samples with SCC >200,000 cells/ml (*n* = 29).

### MALDI-TOF spectra acquisition

After thawing, skimmed milk fractions were centrifuged at 20,000 g for 20 min at + 4°C to remove cell debris and bacteria. Then, each sample was diluted 1:100 with ultrapure water (20) and mixed (1:1) with a matrix solution composed of sinapinic acid (Bruker Daltonics, Bremen, Germany) saturated in 50% *v/v* acetonitrile containing 0.1% trifluoroacetic acid (Merck Darmstadt, Germany). An aliquot (0.5 μl) of each sample was spotted in triplicate on an MSP 96 target ground steel BC (Bruker Daltonics), previously overlaid with a thin layer of sinapinic acid saturated in 100% ethanol (Merck), and allowed to dry for 10 min at room temperature. Spectral profiles were acquired using a MALDI-TOF Microflex LRF mass spectrometer (Bruker Daltonics) equipped with the FlexControl (v. 3.4) software (Bruker Daltonics). Spectra were recorded in the positive linear ion mode within the mass range 4,000–20,000 m/z (laser frequency 20 Hz; ion source 1 voltage, 19.53 kV; ion source 2 voltage, 18.12 kV; lens voltage, 8.12 kV). Three independent spectra for each sample (500 shots each at random positions on the same target place, for spectrum) were manually collected, externally calibrated by the Protein Calibration Standard 1(Bruker Daltonics) and subsequently analyzed.

### Data analysis and model generation

The analysis of all MALDI-TOF-MS data was performed through the ClinProTools software (version 3.0, Bruker Daltonics). The parameters of raw data pre-treatment were set as follows: total average spectra calculation with a resolution equal to 300, baseline subtraction by the top hat baseline algorithm with 10% minimal baseline width, data smoothing by the Savitzky Golay algorithm (width, 4 m/z; smoothing cycles, 20). Recalibration with 1,000 ppm maximal peak shift and 10% match to calibrant peaks was selected. Peak picking was based on the total average spectrum (signal-to-noise threshold, 4) and the maximal peak number was set to 50. Pretreated data were then subjected to visualization and statistical analysis. Normal distribution was checked through the Anderson–Darling test. Peaks with statistically significant differences of signal intensity between the two groups were identified by the Student's *t*-test (for normal variable distribution) or the Wilcoxon test (for non-normal variable distribution). Differences were considered statistically significant when the two-sided *p* < 0.05. The receiver operating characteristic (ROC) curve was calculated for the putative differential peaks. The classification algorithms quick classifier (QC), genetic algorithm (GA), and supervised neural network (SNN) were used to generate the respective models. The accuracy of the class prediction models was evaluated through the calculation of cross-validation and recognition capability. Cross-validation is a measure of the reliability of a model that predicts its future behavior for a given data set and under a given parameterization. Recognition capability describes the performance of an algorithm, intended as the proper classification of a given data set.

## Results

All the milk samples were collected from clinically healthy cows. The microbiological test could not be performed on 19 out of 120 milk samples (Supplementary Table 1). The results of the bacteriological analyses for the routine check of milk quality are summarized in Table 1. In particular, in group H, 3 out of 12 samples microbiologically tested (25%) were positive for *Streptococcus uberis*, and 2 out of 12 samples (17%) were positive for *Staphylococcus* spp; seven samples showed no positivity to any of the tested microorganisms. *Staphylococcus* spp were the only bacteria found in 23 out of 89 microbiologically tested (26%) milk samples from group L (SCC < 200,000 cells/ml).

**Table 1:** Results (number and percentage) of routinary bacteriological analyses on milk samples from group L (*n* = 89) and H (*n* = 12).

	**L**	**H**
*Staphylococcus* spp.	23 (26%)	2 (17%)
*Streptococcus uberis*	ND	3 (25%)
Negative	66 (74%)	7 (58%)

ND, Not detected.

**Figure 1:**
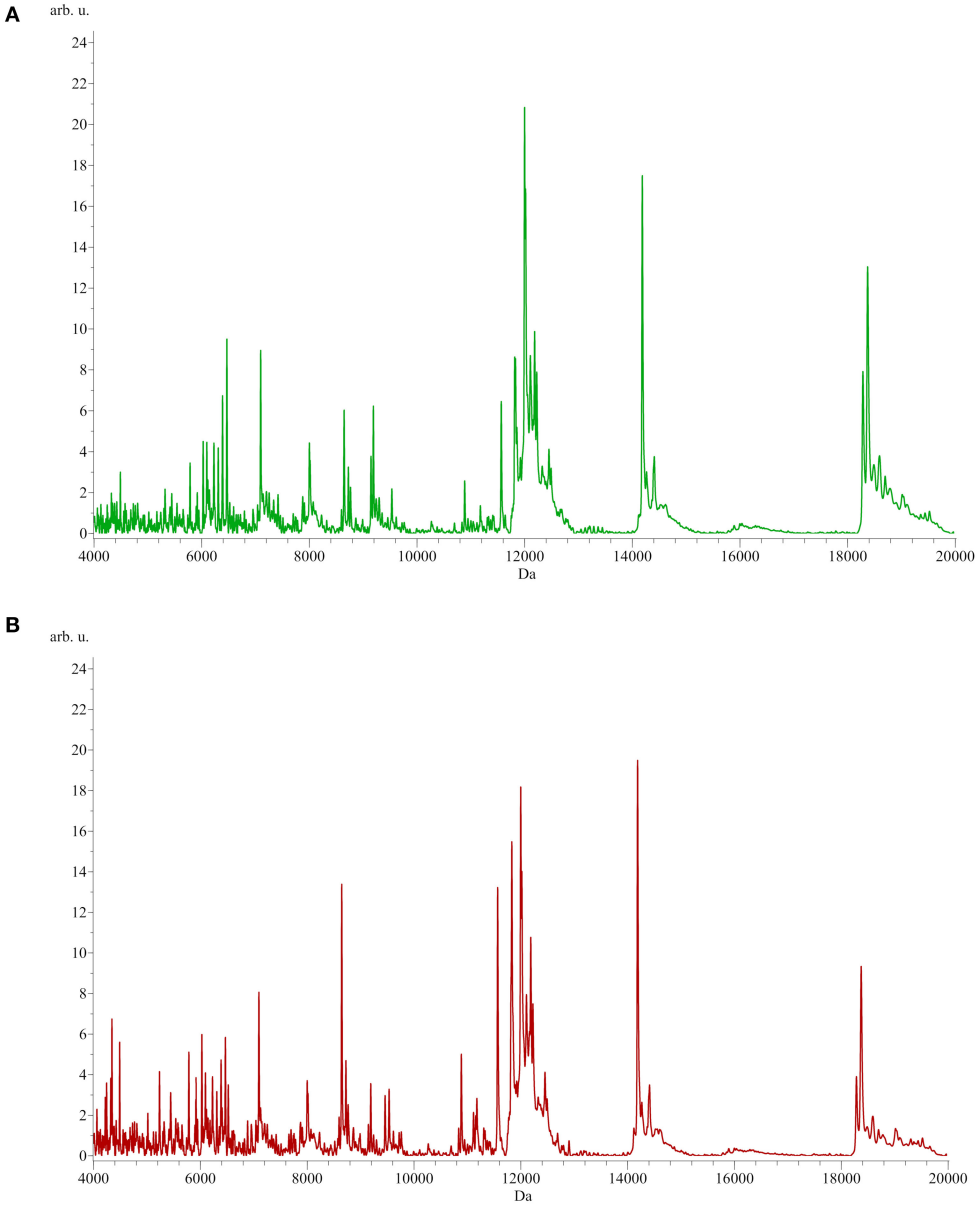
Average MALDI-TOF mass spectra of milk samples from group L [SCC < 200,000 cells/ml, **(A)** green] and H [SCC > 200,000 cells/ml, **(B)** red]. x-axis: m/z, y-axis: arbitrary units of intensity.

**Figure 2:**
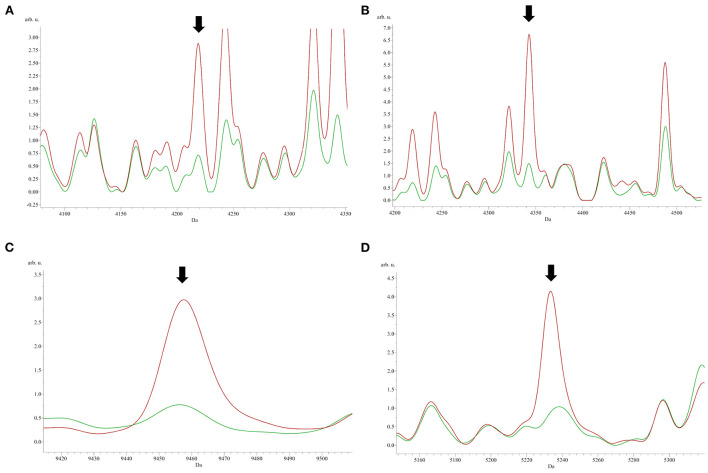
The most significant peaks [**(A)** 4,218.2 m/z; **(B)** 4,342.98 m/z] sorted by the *p*-value of the Wilcoxon test, and the two peaks [**(C)** 9,458.16 m/z; **(D)** 5,234.46 m/z] with fold change values higher than 3 between group L (green) and H (red).

**Figure 3:**
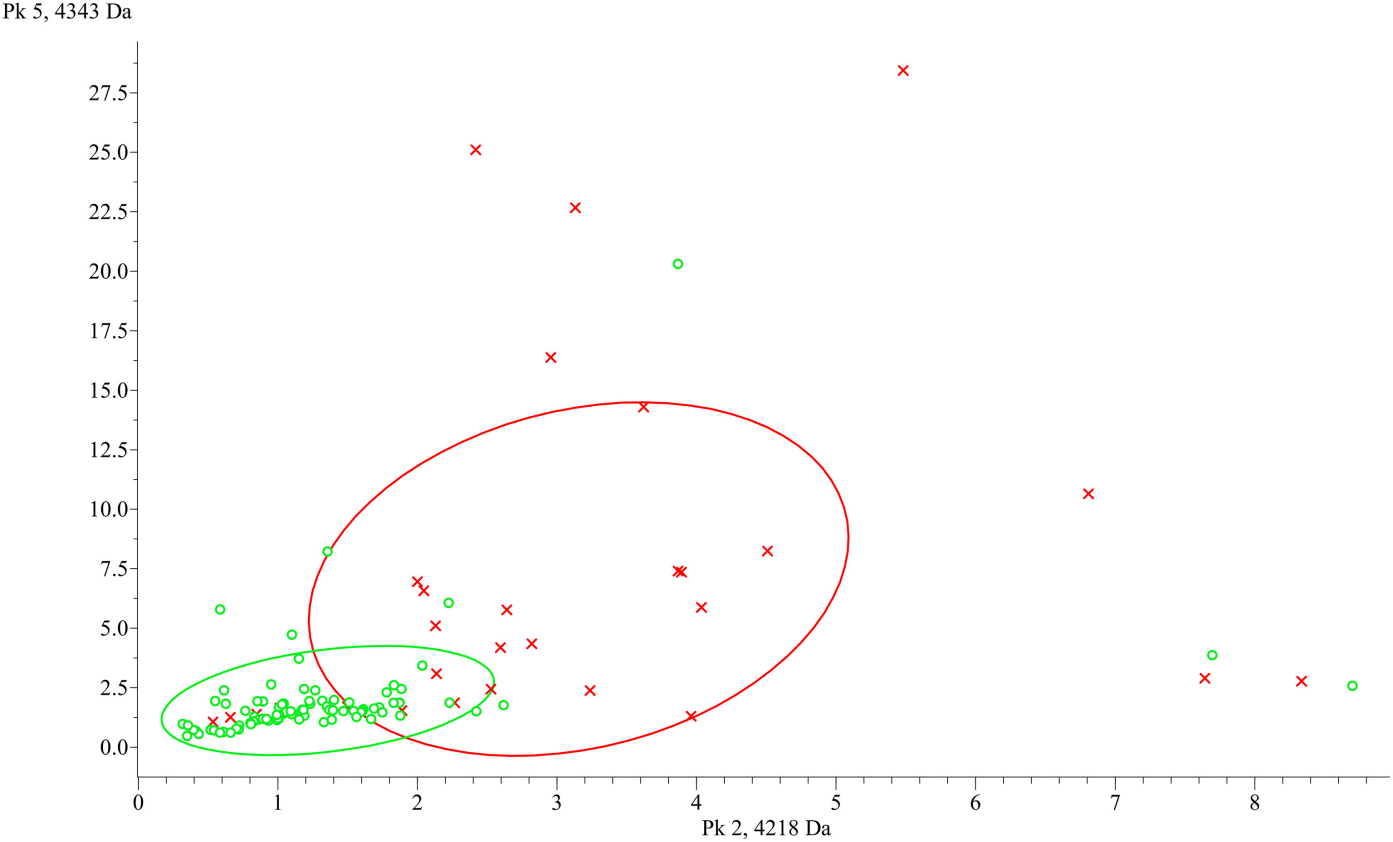
ClinProTools 2D distribution of the two most discriminant peaks between group L (green) and H (red). x-axis: 4,218.2 m/z, y-axis: 4,342.98 m/z.

To identify peptide markers associated with the subclinical mastitis, based on the SCC level, milk samples from groups L and H were analyzed by MALDI-TOF MS in linear mode. The respective spectral profile signatures were generated by acquiring the mass spectra of each sample in triplicate to guarantee reproducibility. The average mass spectra of groups L and H are depicted in Figure 1. Based on a signal-to-noise ratio intensity beyond 4:1, at most 50 peaks for each mass spectra were identified. Significant differences between the two groups were detected according to the signal intensity of peaks. After filtering noise signals and recalibration, 24 peaks exhibited significantly different intensity between group L and H (*p* < 0.05, Wilcoxon test) (Table 2). In particular, 18 and 6 average signals displayed increased or reduced intensity in group H compared to group L, respectively. Among them, 6 peaks showed a fold change (FC) value lower than −2 or higher than +2 between the two groups. In addition to the two most statistically significant average signals (4,218.2 and 4,342.98 m/z), other two peaks resulted as predominantly discriminant, with a FC value higher than 3 (9,458.16, 5,234.98 m/z) (Figure 2). The ROC curve of the two top scored peaks had AUC values of 0.848896 and 0.822549, respectively. These results were used to generate the 2D peak distribution of all samples according to the 4,218.2 and 4,342.98 m/z average signals (Figure 3), which showed a good discrimination between the two groups. Moreover, based on the visual inspection of the average mass spectra, it emerged that signals around 18,000 m/z values show a lower intensity in group H than in group L; on the other hand, peaks around 5,000 m/z have an increased intensity in group H compared to group L.

**Table 2:** Mass signals and corresponding intensity values (mean ± SD) of peaks significantly different between group H (SCC > 200,000 cells/ml) and L (SCC < 200,000 cells/ml).

**Mass** **(*m/z*)**	***p-*value W**	***p*-value AD**	**Group H (mean ±SD)**	**Group L** **(mean ±SD)**	**Fold change**	**AUC**
4,218.2	< 0.000001	< 0.000001	3.49 ± 2.14	1.5 ± 1.32	2.33	0.848896
4,342.98	0.00000392	< 0.000001	7.82 ± 8.23	2.17 ± 2.54	3.60	0.822549
9,458.16	0.00000392	< 0.000001	3.24 ± 2.69	1.01 ± 0.44	3.21	0.820612
5,234.46	0.00000868	0	4.64 ± 5.5	1.47 ± 0.67	3.16	0.807826
11,567.15	0.0000639	0.00000142	13.36 ± 6.63	6.88 ± 5.35	1.94	0.77993
5,916.53	0.000284	< 0.000001	4.2 ± 2.41	2.33 ± 1.38	1.80	0.757071
11,829.8	0.000287	0.0479	16.35 ± 6.63	10.44 ± 5.73	1.57	0.754746
4,244.13	0.0004	< 0.000001	4.46 ± 3.14	2.31 ± 1.64	1.93	0.74506
18,480.68	0.0004	0.000052	1.92 ± 1.57	3.91 ± 2.53	−2.04	0.746997
6,520.66	0.0004	< 0.000001	3.83 ± 2.8	2.01 ± 0.63	1.91	0.744673
4,488	0.00055	< 0.000001	6.1 ± 3.67	3.51 ± 2.05	1.74	0.738473
11,175.67	0.00198	< 0.000001	3.46 ± 3.3	1.68 ± 1.46	2.06	0.716776
18,701.32	0.00247	0.000014	1.56 ± 1.4	2.92 ± 2.05	−1.87	0.71174
5,783.12	0.00302	0.0453	5.41 ± 2.18	3.82 ± 1.94	1.42	0.70709
4,062.13	0.00352	< 0.000001	2.75 ± 1.86	1.94 ± 1.04	1.42	0.703216
18,589.31	0.00567	< 0.000001	2.37 ± 2.31	4.11 ± 3.09	−1.73	0.69353
148,781.2	0.00581	< 0.000001	1.33 ± 1.12	2.4 ± 1.77	−1.8	0.69198
8,646.27	0.00749	< 0.000001	12.8 ± 11.84	6.77 ± 7.41	1.89	0.686168
10,886.8	0.0111	< 0.000001	4.92 ± 3.68	2.93 ± 2.94	1.68	0.677644
9,532.66	0.0168	0.00000125	3.49 ± 1.9	2.44 ± 1.03	1.43	0.667958
5,443.41	0.0168	< 0.000001	3.29 ± 1.9	2.31 ± 1.28	1.42	0.667183
9,189.72	0.0412	< 0.000001	4.21 ± 4.55	6.47 ± 5.83	−1.54	0.646649
6,026.78	0.0485	0.000327	6.35 ± 3.1	4.91 ± 2.13	1.29	0.640837
7,250.85	0.0485	0.0448	1.97 ± 0.58	2.25 ± 0.53	−1.14	0.641612

W, Wilcoxon test; AD, Anderson-Darling test; SD, standard deviation.

Fold change and area under the ROC curve (AUC) values are also reported.

Finally, classification models were generated through three different algorithms (GA, SNN, and QC) and evaluated according to their cross-validation and recognition capability parameters (Table 3). The peaks used by the algorithms for the model generation are reported in Supplementary Table 2. The QC algorithm considered a total of 9 peaks, while the GA and SNN algorithms generated the models by using 20 and 1 peaks, respectively. The performance in discriminating between group H and group L were similar between QC and GA algorithm. QC is characterized by moderate values of both recognition capability (79.91%) and cross-validation value (71.74%). The performance of the model generated by the GA algorithm showed an increased recognition capability (81.03%), but lower cross-validation value (68.17%) than QC. The SNN algorithm resulted as less efficient in properly classifying the samples, with low recognition capability (50.56%) and cross-validation (71.27%) values.

**Table 3:** Cross-validation and recognition capability calculated for each generated model in order to determine a common signature among spectra of group H and group L.

**Algorithm**	**Generated peaks**	**Cross-validation (%)**	**Recognition capability (%)**
GA	20	68.17	81.03
SNN	1	71.27	50.56
QC	9	71.74	79.91

QC, Quick classifier; GA, genetic algorithm, SNN, supervised neural network.

## Discussion

Bovine mastitis is one of the main challenges that farmers and veterinarians routinely have to face in dairy farming. Moreover, the spread of antimicrobial resistance and the recent entering into force of the new European regulation about the use of veterinary medicinal products amplified the need for the implementation of diagnostic tools to early detect subclinical mastitis, to reduce/avoid antimicrobial treatments. In this study, individual milk samples were collected from a total of 120 primiparous Holstein Friesian cows during routinely mastitis screening tests and subjected to MALDI-TOF MS analysis to detect peptide/protein profiles suggestive of the subclinical form of the disease. Indeed, bovine mastitis is able to modify the milk proteome and several proteins have been reported to be differently abundant during mammary gland inflammation (12). To compare healthy subjects with cows potentially affected by subclinical mastitis, the milk samples were classified according to the SCC measurement, setting the 200,000 cell/ml value as a threshold.

In our study, the functional features analyses of milk showed that a total of 23 samples with SCC lower than 200,000 cells/ml were positive for *Staphylococcus spp*. Such result is in line with other studies reporting low SCC levels in primiparous cows and/or positive for non-aureus staphylococci (22, 23). However, recent studies have also described less pathogenic *Staphylococcus* strains, whose virulence factors could induce less severe mastitis or a delayed SCC increase (24, 25). Indeed, the host-pathogen interaction varies according to the stage of mastitis and the arousal of the inflammatory response depends on the responsiveness of the host immune system (24, 26, 27). On the other hand, 7 milk samples presented negative results to the microbiological analysis, but SCC higher than 200,000 cells/ml. Such discrepancy can be explained by the well-known limitations of the microbiological assays (4, 28, 29). In addition, also *Mycoplasma* spp. should be considered, due to difficulties in its cultivation and isolation procedures (4).

The results of the milk protein profiling showed statistically significant differences between samples with high SCC level (> 200,000 cell/ml, group H) and samples with low SCC level (< 200,000 cell/ml, group L). In particular, 24 polypeptides/proteins are differentially abundant, with both increasing and decreasing levels in group H compared to group L, considered as healthy. Thus, a panel of several protein markers can be further investigated and proposed as a complementary diagnostic tool for subclinical mastitis, increasing the recognition capability of standard methods. Considering the ROC curve of the two top scored peaks (4,218.2 and 4,342.98 m/z), our results are promising since AUC values higher than 0.8 represent an acceptable accuracy in the differentiation of the two groups. Moreover, other two peaks presented FC values higher than 3 suggesting a difference in the protein content between the two groups. Moreover, the 2D peak distribution plot built on the two top scored peaks show a clear separation between the two groups. Anyway, some samples are located in the opposite cluster and this could be due to the differences in the microbiological results, which include positivity to *Staphylococcus* spp. and *Streptococcus uberis*, as well as negative isolations. It is well-known that etiological agents can differently influence mastitis pathogenesis (30, 31), and differences in the milk peptidome between *Escherichia coli* and *S. aureus* mastitis have already been reported (16). Therefore, an influence of the microbiological agents involved in the inflammatory process could not be excluded. The possibility to generate a robust model to discriminate healthy and mastitic milk was explored through three different algorithms. Considering the data of our study and the specific features of the algorithm, the best performances were exhibited by the QC algorithm. Indeed, it works by classifying the different spectra according to the statistical *p*-values at certain peak positions (32). The other two algorithms, SNN and GA, resulted as less suitable for mastitis discrimination, due to its multifactorial features. SNN tries to identify some prototypical spectra for each class and GA is an evolutionary algorithm influenced by mutations, crossover and selection phenomena (32).

Different mass spectrometry-based approaches were used in previous studies to investigate the role of the milk proteome or peptidome as a source of potential biomarkers of mastitis (13, 14, 16, 33). In the present study an untargeted MALDI-TOF MS profiling was applied, which has the advantage, compared to other proteomic techniques, to provide reliable results in a cost and time effective way. Previous studies have already investigated the application of MALDI-TOF MS to the identification of protein mastitis markers (34, 35). However, the differences in applied methods and in the animal species and breed investigated do not enable a meaningful comparison with our results. However, taken together all these studies support the MALDI-TOF MS milk profiling as a possible application in the early diagnosis of subclinical mastitis of dairy animals.

## Conclusion

In this study, milk samples with SCC higher and lower than 200,000 cells/ml were compared through a MALDI-TOF MS approach. The results showed that the milk protein profiles significantly vary according to SCC level and several polypeptides/proteins are differently abundant between the two groups. SCC testing still remains the most convenient and preferable method for udder health monitoring; nevertheless, the association of SCC measurement and milk protein profiling by MALDI-TOF MS may facilitate earlier identification of subclinical forms of mastitis. Further studies with a larger and well-characterized sample set are envisaged to confirm our preliminary findings on MALDI-TOF MS profiling of skim milk for mastitis detection.

## Data availability statement

The original contributions presented in the study are included in the article/Supplementary material, further inquiries can be directed to the corresponding author. Raw spectra acquired with MALDI-TOF MS are uploaded in the open access repository in https://zenodo.org/ accessible at http://doi.org/10.5281/zenodo.7177944.

## Ethics statement

Ethical review and approval was not required for the animal study because milk was collected during the routine monitoring of milk quality on farms. Animals were managed according to the local farm-production practices. All manipulations were performed kindly to avoid animal distress. Written informed consent was obtained from the owners for the participation of their animals in this study.

## Author contributions

FG and FC conceived, designed the study, and revised and edited the final version of the manuscript. MC and SD performed the analyses and wrote the original draft. MC, SD, and FG performed data curation and statistical analysis. FG, FC, and PS supervised the study. FC and PS acquired the funding. All authors contributed to the article and approved the submitted version.

## Funding

This research was funded by TECH4MILK–Tecnologie e soluzioni innovative al servizio della filiera del latte piemontese, Grant Number 333-183.

## Conflict of interest

The authors declare that the research was conducted in the absence of any commercial or financial relationships that could be construed as a potential conflict of interest.

## Publisher's note

All claims expressed in this article are solely those of the authors and do not necessarily represent those of their affiliated organizations, or those of the publisher, the editors and the reviewers. Any product that may be evaluated in this article, or claim that may be made by its manufacturer, is not guaranteed or endorsed by the publisher.
