# Supplementary material for: MALDI-TOF mass spectrometry profiling of bovine skim milk for subclinical mastitis detection
Source: Front Vet Sci. 2022 Dec 1;9:1009928. doi: 10.3389/fvets.2022.1009928 (PMC9753775; doi:10.3389/fvets.2022.1009928)
Supplement: Supplementary file 1 [file Table_1.docx]

Table S1. Sampling of milk randomly collected 120 primiparous Friesian cows coming from twelve dairy farms in the province of Cuneo, Piedmont, Italy during December 2020 – February 2021,July – September 2021 and September-October 2022. Successively, the samples were divided into two groups based on SCC values: group L with SCC less than 200,000 cells/ml and group H with SCC greater than 200,000 cells/ml.

| ID  sample | ID  farm | Calving  date | Sampling  date | Days  in milk | SCC  (cells/ml) | Microbiology |
| --- | --- | --- | --- | --- | --- | --- |
| 1 | 1 | 18/8/2020 | 2/12/2020 | 106 | 30,000 | Negative |
| 2 | 1 | 7/8/2020 | 2/12/2020 | 117 | 13,000 | Negative |
| 3 | 1 | 5/8/2020 | 2/12/2020 | 119 | 6,000 | Negative |
| 4 | 1 | 16/8/2020 | 2/12/2020 | 108 | 23,000 | Negative |
| 5 | 1 | 12/9/2020 | 2/12/2020 | 81 | 7,000 | Negative |
| 6 | 1 | 7/8/2020 | 2/12/2020 | 117 | 10,000 | Negative |
| 7 | 2 | 24/10/2020 | 17/12/2020 | 54 | 21,000 | Negative |
| 8 | 2 | 8/11/2020 | 17/12/2020 | 39 | 17,000 | *Staphylococcus* spp. |
| 9 | 2 | 22/9/2020 | 17/12/2020 | 86 | 32,000 | *Staphylococcus* spp. |
| 10 | 2 | 15/10/2020 | 17/12/2020 | 63 | 8,000 | Negative |
| 11 | 3 | 2/7/2020 | 12/1/2021 | 194 | 331,000 | Negative |
| 12 | 3 | 15/6/2020 | 12/1/2021 | 211 | 27,000 | *Staphylococcus* spp. |
| 13 | 3 | 27/10/2020 | 12/1/2021 | 77 | 2,000 | Negative |
| 14 | 3 | 13/12/2020 | 12/1/2021 | 30 | 21,000 | Negative |
| 15 | 4 | 28/10/2020 | 25/1/2021 | 89 | 11,000 | Negative |
| 16 | 4 | 9/1/2021 | 25/1/2021 | 16 | 8,000 | *Staphylococcus* spp. |
| 17 | 4 | NA | 25/1/2021 | NA | 155,000 | *Staphylococcus* spp. |
| 18 | 5 | 23/1/2021 | 28/1/2021 | 5 | 31,000 | Negative |
| 19 | 5 | 16/1/2021 | 28/1/2021 | 12 | 18,000 | *Staphylococcus* spp. |
| 20 | 5 | 25/12/2020 | 28/1/2021 | 34 | 21,000 | *Staphylococcus* spp. |
| 21 | 5 | 8/11/2020 | 28/1/2021 | 81 | 5,000 | Negative |
| 22 | 5 | 14/12/2020 | 28/1/2021 | 45 | 8,000 | Negative |
| 23 | 5 | 30/12/2020 | 28/1/2021 | 29 | 10,000 | Negative |
| 24 | 5 | 2/9/2020 | 28/1/2021 | 148 | 32,000 | *Staphylococcus* spp. |
| 25 | 5 | 8/12/2020 | 28/1/2021 | 51 | 23,000 | Negative |
| 26 | 5 | 3/12/2020 | 28/1/2021 | 56 | 18,000 | *Staphylococcus* spp. |
| 27 | 5 | 8/11/2020 | 28/1/2021 | 81 | 6,000 | Negative |
| 28 | 6 | 3/8/2020 | 4/2/2021 | 185 | 102,000 | Negative |
| 29 | 6 | 19/8/2020 | 4/2/2021 | 169 | 6,359,000 | Negative |
| 30 | 6 | 3/8/2020 | 4/2/2021 | 185 | 69,000 | Negative |
| 31 | 6 | 17/8/2020 | 4/2/2021 | 171 | 53,000 | *Staphylococcus* spp. |
| 32 | 6 | 26/8/2020 | 4/2/2021 | 162 | 47,000 | Negative |
| 33 | 7 | 15/1/2021 | 5/2/2021 | 21 | 15,000 | Negative |
| 34 | 7 | 1/1/2021 | 5/2/2021 | 35 | 10,000 | Negative |
| 35 | 7 | 25/10/2020 | 5/2/2021 | 103 | 18,000 | Negative |
| 36 | 7 | 29/8/2020 | 5/2/2021 | 160 | 17,000 | Negative |
| 37 | 8 | 20/7/2020 | 11/2/2021 | 206 | 66,000 | Negative |
| 38 | 8 | 19/8/2020 | 11/2/2021 | 176 | 66,000 | Negative |
| 39 | 8 | 3/10/2020 | 11/2/2021 | 131 | 47,000 | Negative |
| 40 | 8 | 1/11/2020 | 11/2/2021 | 102 | 7,000 | Negative |
| 41 | 8 | 23/11/2020 | 11/2/2021 | 80 | 12,000 | Negative |
| 42 | 8 | 11/7/2020 | 11/2/2021 | 215 | 36,000 | Negative |
| 43 | 8 | 1/10/2020 | 11/2/2021 | 133 | 94,000 | Negative |
| 44 | 8 | 5/7/2020 | 11/2/2021 | 221 | 133,000 | Negative |
| 45 | 9 | 7/7/2020 | 17/2/2021 | 225 | 25,000 | Negative |
| 46 | 9 | 4/9/2020 | 17/2/2021 | 166 | 443,000 | *Streptococcus* uberis |
| 47 | 9 | 4/10/2020 | 17/2/2021 | 136 | 14,000 | Negative |
| 48 | 9 | 10/10/2020 | 17/2/2021 | 130 | 30,000 | *Staphylococcus* spp. |
| 49 | 9 | 3/10/2020 | 17/2/2021 | 137 | 122,000 | *Staphylococcus* spp. |
| 50 | 10 | 20/9/2020 | 25/2/2021 | 158 | 8,000 | Negative |
| 51 | 10 | 27/11/2020 | 25/2/2021 | 90 | 548,000 | Negative |
| 52 | 10 | 3/8/2020 | 25/2/2021 | 206 | 138,000 | Negative |
| 53 | 10 | 10/8/2020 | 25/2/2021 | 199 | 107,000 | *Staphylococcus* spp. |
| 54 | 10 | 23/8/2020 | 25/2/2021 | 186 | 8,000 | *Staphylococcus* spp. |
| 55 | 10 | 19/9/2020 | 25/2/2021 | 159 | 1,213,000 | *Staphylococcus* spp. |
| 56 | 10 | 3/8/2020 | 25/2/2021 | 206 | 189,000 | Negative |
| 57 | 10 | 24/11/2020 | 25/2/2021 | 93 | 44,000 | *Staphylococcus* spp. |
| 58 | 8 | 20/7/2020 | 8/7/2021 | 353 | 117,000 | Negative |
| 59 | 8 | 3/10/2020 | 8/7/2021 | 278 | 43,000 | Negative |
| 60 | 8 | 1/11/2020 | 8/7/2021 | 249 | 38,000 | Negative |
| 61 | 8 | 23/11/2020 | 8/7/2021 | 227 | 95,000 | Negative |
| 62 | 8 | 11/7/2020 | 8/7/2021 | 362 | 104,000 | *Staphylococcus* spp. |
| 63 | 8 | 5/7/2020 | 8/7/2021 | 368 | 25,000 | Negative |
| 64 | 3 | 15/6/2020 | 9/7/2021 | 389 | 39,000 | Negative |
| 65 | 3 | 2/11/2020 | 9/7/2021 | 249 | 16,000 | Negative |
| 66 | 3 | 27/10/2020 | 9/7/2021 | 255 | 6,000 | Negative |
| 67 | 3 | 13/12/2020 | 9/7/2021 | 208 | 4,000 | Negative |
| 68 | 1 | 5/8/2020 | 20/7/2021 | 349 | 150,000 | Negative |
| 69 | 1 | 16/8/2020 | 20/7/2021 | 338 | 3,769,000 | Negative |
| 70 | 1 | 7/8/2020 | 20/7/2021 | 347 | 4,609,000 | *Streptococcus* *uberis* |
| 71 | 9 | 4/9/2020 | 27/7/2021 | 326 | 4,827,000 | *Streptococcus* *uberis* |
| 72 | 9 | 4/10/2020 | 27/7/2021 | 296 | 48,000 | Negative |
| 73 | 9 | 3/10/2020 | 27/7/2021 | 297 | 51,000 | Negative |
| 74 | 2 | 8/11/2020 | 28/7/2021 | 262 | 106,000 | Negative |
| 75 | 2 | 16/11/2020 | 28/7/2021 | 254 | 39,000 | Negative |
| 76 | 2 | 15/10/2020 | 28/7/2021 | 286 | 24,000 | Negative |
| 77 | 2 | 16/11/2020 | 28/7/2021 | 254 | 7,000 | Negative |
| 78 | 5 | 23/1/2021 | 30/7/2021 | 188 | 224,000 | Negative |
| 79 | 5 | 16/1/2021 | 30/7/2021 | 195 | 8,000 | *Staphylococcus* spp. |
| 80 | 5 | 25/12/2020 | 30/7/2021 | 217 | 62,000 | *Staphylococcus* spp. |
| 81 | 5 | 8/11/2020 | 30/7/2021 | 264 | 19,000 | Negative |
| 82 | 5 | 14/12/2020 | 30/7/2021 | 228 | 210,000 | Negative |
| 83 | 5 | 2/9/2020 | 30/7/2021 | 331 | 27,000 | *Staphylococcus* spp. |
| 84 | 5 | 8/12/2020 | 30/7/2021 | 234 | 76,000 | *Staphylococcus* spp. |
| 85 | 5 | 8/11/2020 | 30/7/2021 | 264 | 9,000 | Negative |
| 86 | 4 | 9/1/2021 | 6/9/2021 | 240 | 6,000 | Negative |
| 87 | 4 | 21/12/2020 | 6/9/2021 | 259 | 20,000 | Negative |
| 88 | 4 | 20/2/2021 | 6/9/2021 | 198 | 46,000 | Negative |
| 89 | 4 | 14/5/2021 | 6/9/2021 | 115 | 642,000 | Negative |
| 90 | 6 | 3/8/2020 | 7/9/2021 | 400 | 827,000 | *Staphylococcus* spp. |
| 91 | 6 | 26/8/2020 | 7/9/2021 | 377 | 178,000 | *Staphylococcus* spp. |
| 92 | 6 | 25/2/2021 | 7/9/2021 | 194 | 43,000 | Negative |
| 93 | 6 | 12/2/2021 | 7/9/2021 | 207 | 14,000 | Negative |
| 94 | 7 | 3/1/2021 | 8/9/2021 | 248 | 13,000 | Negative |
| 95 | 7 | 15/1/2021 | 8/9/2021 | 236 | 7,000 | Negative |
| 96 | 7 | 1/1/2021 | 8/9/2021 | 250 | 31,000 | Negative |
| 97 | 7 | 25/10/2020 | 8/9/2021 | 318 | 21,000 | Negative |
| 98 | 10 | 20/9/2020 | 27/9/2021 | 372 | 171,000 | *Staphylococcus* spp. |
| 99 | 10 | 27/11/2020 | 27/9/2021 | 304 | 6,000 | Negative |
| 100 | 10 | 3/8/2020 | 27/9/2021 | 420 | 83,000 | Negative |
| 101 | 10 | 24/11/2020 | 27/9/2021 | 307 | 21,000 | *Staphylococcus* spp. |
| 102 | 11 | 13/04/2022 | 19/09/2022 | 159 | 216,000 | NA |
| 103 | 11 | NA | 19/09/2022 | NA | 141,000 | NA |
| 104 | 11 | 23/06/2022 | 19/09/2022 | 88 | 229,000 | NA |
| 105 | 11 | 05/12/2021 | 19/09/2022 | 288 | 716,000 | NA |
| 106 | 11 | 27/09/2021 | 19/09/2022 | 357 | 225,000 | NA |
| 107 | 11 | NA | 19/09/2022 | NA | 11,000 | NA |
| 108 | 11 | 09/11/2021 | 19/09/2022 | 314 | 214,000 | NA |
| 109 | 11 | 10/11/2021 | 19/09/2022 | 313 | 1,062,000 | NA |
| 110 | 11 | 20/02/2022 | 19/09/2022 | 211 | 301,000 | NA |
| 111 | 11 | 10/11/2021 | 19/09/2022 | 313 | 208,000 | NA |
| 112 | 11 | 30/11/2021 | 28/09/2022 | 302 | 777,000 | NA |
| 113 | 11 | 18/07/2022 | 28/09/2022 | 72 | 2,298,000 | NA |
| 114 | 11 | 27/09/2021 | 28/09/2022 | 366 | 201,000 | NA |
| 115 | 11 | 29/01/2022 | 28/09/2022 | 242 | 325,000 | NA |
| 116 | 11 | 24/02/2022 | 28/09/2022 | 216 | 3,871,000 | NA |
| 117 | 12 | 25/04/2022 | 05/10/2022 | 163 | 308,000 | NA |
| 118 | 12 | 28/01/2022 | 05/10/2022 | 250 | 360,000 | NA |
| 119 | 12 | 25/04/2022 | 05/10/2022 | 163 | 414,000 | NA |
| 120 | 12 | 19/03/2022 | 05/10/2022 | 200 | 406,000 | NA |

NA: not available data
