# Supplementary material for: MALDI-TOF mass spectrometry profiling of bovine skim milk for subclinical mastitis detection
Source: Front Vet Sci. 2022 Dec 1;9:1009928. doi: 10.3389/fvets.2022.1009928 (PMC9753775; doi:10.3389/fvets.2022.1009928)
Supplement: Supplementary file 2 [file Table_2.docx]

Table S2. Peaks used by the algorithm for model generation in ClinProTools.

| Algorithm | Index | Mass (m/z) |
| --- | --- | --- |
| GA | 36 | 11,829.8 |
|  | 32 | 9,6532.66 |
|  | 39 | 12,108.42 |
|  | 26 | 8,646.27 |
|  | 35 | 11,567.15 |
|  | 23 | 7,250.85 |
|  | 14 | 6,094.53 |
|  | 12 | 5,916.53 |
|  | 3 | 4,244.13 |
|  | 22 | 7,200.74 |
|  | 15 | 6,140.91 |
|  | 17 | 6,307.32 |
|  | 29 | 9,145.23 |
|  | 31 | 9,458.16 |
|  | 20 | 6,520.66 |
|  | 33 | 10,886.8 |
|  | 13 | 6,026.78 |
|  | 18 | 6,386.84 |
|  | 49 | 18,701.32 |
|  | 45 | 18,289.92 |
| QC | 2 | 4,218.2 |
|  | 3 | 4,244.13 |
|  | 5 | 4,342.98 |
|  | 8 | 5,234.46 |
|  | 12 | 5,916.53 |
|  | 20 | 6,520.66 |
|  | 31 | 9,458.16 |
|  | 35 | 11,567.15 |
|  | 36 | 11,829.8 |
| SNN | 30 | 9,189.72 |
